# High dietary salt intake correlates with modulated Th17-Treg cell balance resulting in enhanced bone loss and impaired bone-microarchitecture in male mice

**DOI:** 10.1038/s41598-018-20896-y

**Published:** 2018-02-06

**Authors:** Hamid Y. Dar, Anjali Singh, Prashant Shukla, Rajaneesh Anupam, Rajesh K. Mondal, Pradyumna K. Mishra, Rupesh K. Srivastava

**Affiliations:** 1 Department of Zoology, School of Biological Sciences, Dr. Harisingh Gour Central University, Sagar, MP 470003 India; 2 Department of Biotechnology, School of Biological Sciences, Dr. Harisingh Gour Central University, Sagar, MP 470003 India; 3 Department of Physics, School of Mathematical and Physical Sciences, Dr. Harisingh Gour Central University, Sagar, MP 470003 India; 4 Department of Microbiology, School of Biological Sciences, Dr. Harisingh Gour Central University, Sagar, MP 470003 India; 5 Department of Molecular Biology, National Institute for Research in Environmental Health, Bhopal, MP 462001 India; 6 Present Address: Department of Biotechnology, All India Institute of Medical Sciences (AIIMS), New Delhi, 110029 India

## Abstract

Osteoporosis is associated with reduced density and quality of bone leading to weakened skeleton thereby increasing the risk of fractures responsible for increased morbidity and mortality. Due to preference for western food style the consumption of salt intake in our diets has increased many folds. High dietary salt intake has recently been linked with induction of Th17 cells along with impairment of Treg cells. Also, Th17 cells have been one of major players in the pathophysiology of various bone pathologies including osteoporosis. We thus hypothesized that high salt diet (HSD) intake would lead to enhanced bone loss by modulating Th17-Treg cell balance. In the present study, we report for the first time that HSD intake in male mice impairs both trabecular and cortical bone microarchitecture along with decreasing the mineral density and heterogeneity of bones. The HSD modulates host immune system and skews Treg-Th17 balance by promoting osteoclastogenic Th17 cells and inhibiting development of anti-osteoclastogenic Treg cells in mice. HSD also enhanced expression of proinflammatory cytokines (IL-6, TNF-α, RANKL and IL-17) and decreased the expression of anti-inflammatory cytokines (IL-10, IFN-γ). Taken together the present study for the first time establishes a strong correlation between high dietary salt intake and bone health via interplay between Th17-Treg cells.

## Introduction

Osteoporosis is an increasingly common chronic condition of bones with more than 200 million affected individuals worldwide^1^. Osteoporosis is associated with reduced density and quality of bone leading to weakened skeleton thereby increasing the risk of fractures, responsible for increased morbidity and mortality^2^. In addition, osteoporosis will take a heavy toll on the economy with an estimated burden of USD 131.5 billion worldwide by 2050^2^. The role of nutrition in bone health has now gained momentum with a number of minerals and vitamins been identified to play a potential role in the prevention of bone diseases, particularly osteoporosis. Different nutrients have been proposed to play key role in bone development, maintenance and bone loss^3^. Thus, diet constitutes one of the main contributory and regulatory factors in bone health^4,5^. Among these, the relevance of dietary calcium to bone mass has been well documented in numerous studies in humans^6^. In contrast, the importance of other essential nutrients including the effect of sodium intake on bone health has not been yet fully elucidated^7,8^. It is well known that high dietary sodium intake decreases renal calcium reabsorption that results in greater urinary calcium excretion. According to World Health Organization (WHO) the recommended daily intake of salt in adults is 5 g/day in adults^9,10^, but with the advent of western diet-style worldwide, the mean daily salt intake has increased to 12 g/day^9–11^. Intake of high salt diet (HSD) leads to increased risks of hypertension, cardiovascular diseases along with impairment of kidneys^12,13^. HSD not only elevates the blood pressure but in long run causes ventricle hypotrophy, proteinuria as well as strokes^14–16^. In this scenario, WHO has set up goal of decreasing the salt intake upto 30% by 2025^12,17^. As the salt war rages on, it has now been also linked to negative effect on bone health, as various recent studies have reported relationship between high salt intake and risk of osteoporosis^7,18^. It has been observed that high dietary salt intake induces hypercalciuria along with greater calcium excretion^19^.

Th17 cells are one of the major cells responsible for enhanced osteoclastogenesis and resulting bone loss by producing higher levels of IL-17, RANKL, TNF-α and lower levels of IFN-γ^20–22^. Whereas, regulatory T cells (Tregs) through their production of IL-10 and TGF-β1 suppress the effector functioning of Th17 cells. Tregs can also lead to suppression of bone loss by inhibiting differentiation of monocytes into osteoclasts under both *in vitro* and *in vivo* conditions^23,24^. Recently it has been reported by Jörg *et al*. that HSD induces the generation of pathogenic Th17 cells in experimental autoimmune encephalomyelitis (EAE)^25^. Also, HSD has been associated with higher incidences of rheumatoid arthritis (RA)^26,27^, along with affecting the innate immune system by enhancing the functioning of proinflammatory macrophages^28,29^. Furthermore, HSD have been reported to impair functioning of Treg cells^30^. Thus, very few studies till date have linked the negative effects of HSD on bone loss, but most importantly no study had ever established the role of T cells (Treg-Th17) in high dietary salt induced bone loss. In our present study, we report for the first time that high dietary salt intake impairs bone-microarchitecture and induces bone loss by modulating Treg-Th17 cell balance. We report that HSD significantly enhances induction of Th17 cells along with simultaneous decrease in the population of Treg cells *in vivo*. This effect of HSD is mediated by enhanced expression of proinflammatory cytokines (IL-6, IL-17, RANKL and TNF-α) and decreased expression of anti-inflammatory cytokines (IL-10, IFN-γ). The present study thus demonstrates the unique role of diet, particularly high salt in bone health; and thus opens up Pandora’s Box for future research in the novel field of “Nutritional Therapeutics”.

## Results

### High dietary salt intake enhances bone loss

**Figure 1:**
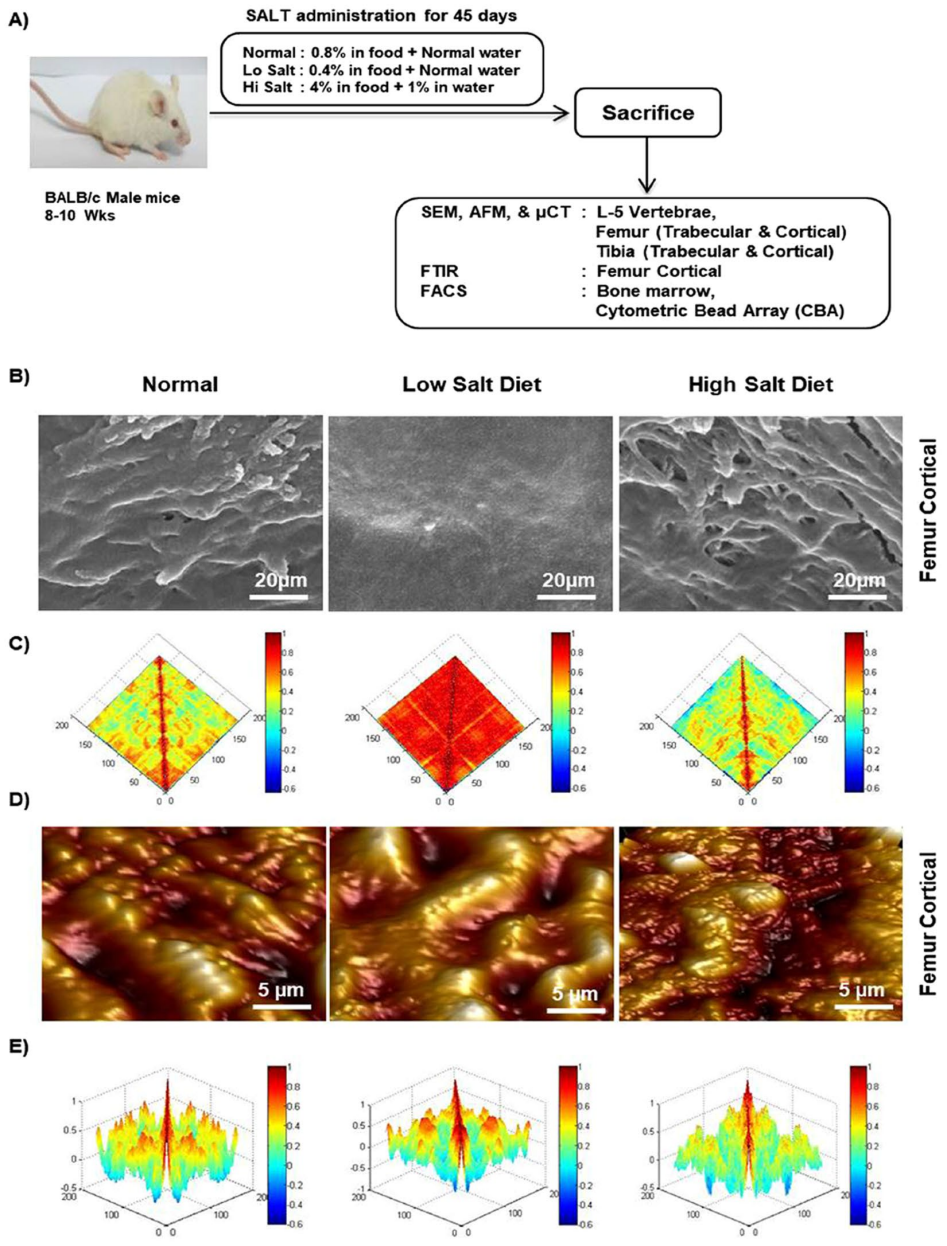
High dietary salt intake enhances bone loss. (**A**) Experimental work plan. Mice were divided into 3 groups. The high salt diet group (HSD) mice received diet containing 4% NaCl in chow plus 1% in drinking water. The low salt diet group (LSD) mice were fed on diet having 0.4% NaCl in chow plus normal drinking water whereas normal mice were given a diet containing 0.8% NaCl in chow plus normal drinking water for 45 days. At the end of experiment mice were sacrificed and tissues (bones+ lymphoid) were analysed. (**B**) 2D SEM images of femur cortical. (**C**) 2D MATLAB analysis of SEM images. (**D**) 3D AFM images of femur cortical. (**E**) 3D MATLAB analysis of AFM images. The above images are representative of one experiment and similar results were obtained in three different experiments with n = 10 mice/group/experiment. (Mouse image courtesy: Hamid Y. Dar).

To determine the effect of high dietary salt intake on bone health we were first interested in looking for different signs of bone remodelling by scanning the surface of bones by scanning electron microscopy (SEM) and atomic force microscopy (AFM). To determine the same we divided the mice in three group’s viz. normal (0.8% in chow + normal drinking water), low salt diet (LSD) (0.4% in chow + normal drinking water) and high salt diet (HSD) intake (4% in chow + 1% drinking water) groups (Fig. 1A) and at day 45 (Supplementary Fig. S1, S2 and S3) mice were sacrificed and femur bones collected for SEM and AFM. SEM images (Fig. 1B) clearly indicated a significantly higher number of resorption pits/lacunae (increased osteoclastogenesis) of various shapes/area on the surface of the bones in HSD group with respect to LSD group. Notably these resorption pits/lacunae were either significantly absent or were in very few numbers in LSD intake group (Fig. 1B). AFM is a superb tool for imaging of bone ultra-structure in a close to physiological state^31^. We thus next performed AFM for a detailed understanding of the influence of dietary salt intake on bone remodelling. As expected our AFM images too complemented our results from SEM by revealing significantly decreased number of scalloped surfaces or pits (Fig. 1D) left behind by the osteoclasts in LSD intake group in comparison to both normal and HSD intake groups.

MATLAB (matrix laboratory) is a multi-paradigm numerical computing technique used in microscopy, biomedical imaging etc. We thus performed MATLAB analysis of both SEM and AFM images to further analyse our data, regarding correlation between bone loss and bone topology. In MATLAB any distortion in the object or absence of identical object reduces the correlation value and is usually represented through colours. It can be clearly concluded from Fig. 1C (2D-SEM) that LSD group has different topology from that of normal and HSD groups which is very much degraded (enhanced osteoclastogenesis). The MATLAB-analysis of SEM images (Fig. 1C) shows this extent of homogeneity observed in the SEM images by red (highest correlation = more bone) and blue (least correlation = less bone). Figure 1D (3D-AFM) further complements our results that LSD group has normal bone topology and high bone content whereas, both normal and HSD groups have bone surface eaten up due to enhanced osteoclastogenesis. In MATLAB-analysis of AFM images (Fig. 1E), red implies height of bone (suppressed osteoclastogenesis) and the representative valleys-blue describe decreased bone height (enhanced osteoclastogenesis). Collectively, our data from both SEM and AFM analysis of bone samples clearly indicate that HSD intake enhances bone loss, whereas LSD intake leads to significant inhibition of bone loss.

### High dietary salt intake impairs lumbar vertebral (LV5) bone micro-architecture

**Figure 2:**
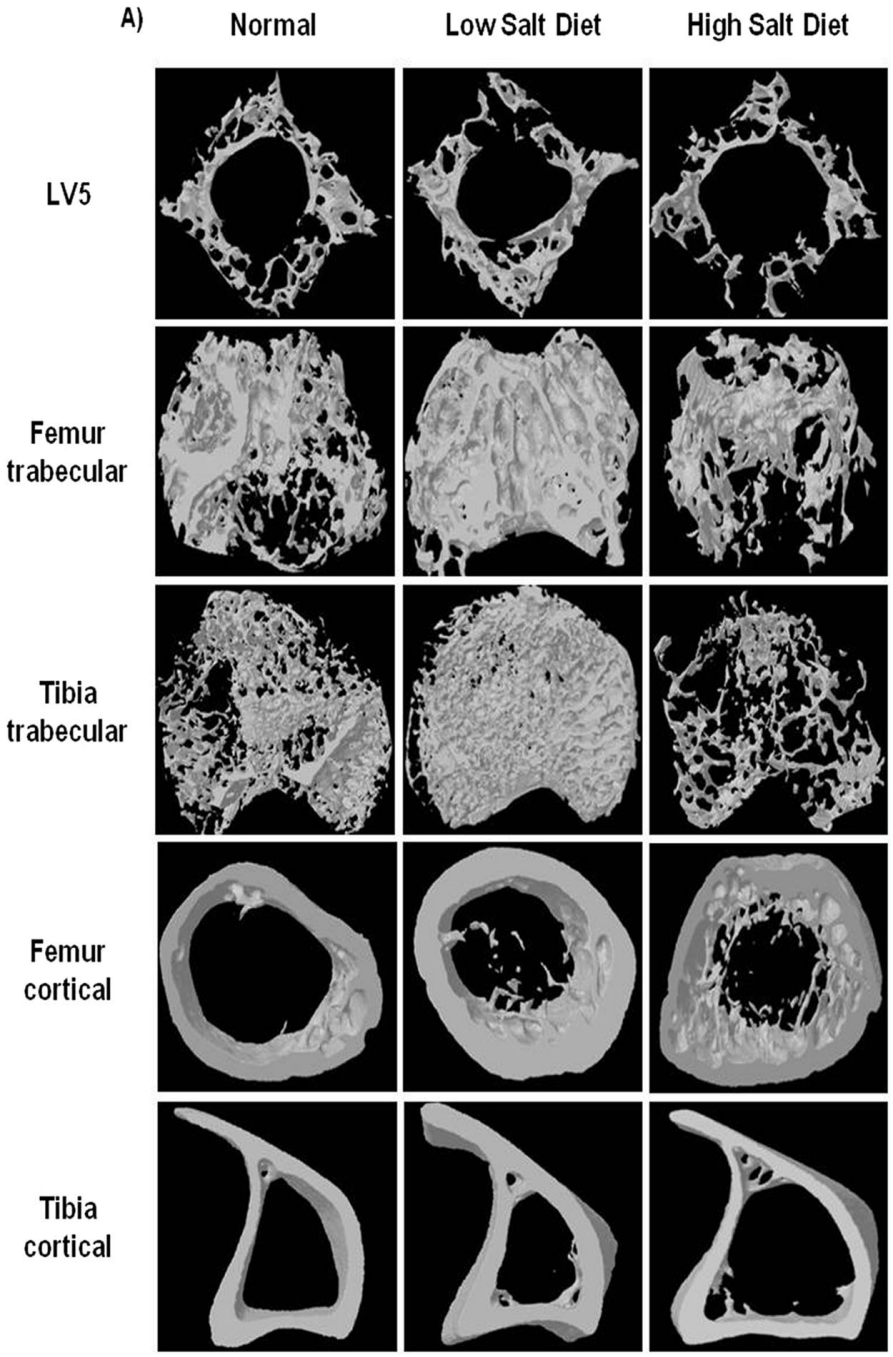
High dietary salt intake reduces trabecular and cortical bone microarchitecture. (**A**) 3-D μCT reconstructions of LV5, femur trabecular, tibia trabecular, femur cortical and tibia cortical of normal, low salt diet (LSD) and high salt diet groups (HSD). The above images are representative of one experiment and similar results were obtained in three different experiments with n = 10 mice/group/experiment.

Moving ahead in our study we were next interested in delineating the effect of HSD on bone microarchitecture. We thus performed µCT (a “gold standard” for evaluation of bone morphology and microarchitecture) for quantitating various bone morphometric and geometric parameters related to bone loss. Since the lumbar vertebrae-5 (LV-5) is considered as one of the best diagnostic parameters to study the effect of bone loss via µCT in osteoporosis, we too analysed the effect of HSD on LV-5 vertebrae. Strikingly, μCT analysis clearly pointed towards a more compact LV-5 architecture in LSD intake group with respect to both normal and HSD intake groups (Fig. 2). We observed that in comparison to LSD intake group, HSD group had decreased bone volume/tissue volume (BV/TV) (p < 0.01), trabecular thickness (Tb. Th) (p < 0.01), trabecular number (Tb. N) (p < 0.01) and connectivity density (Conn. Den.) (p < 0.01) (Table 1). On the other hand in comparison to LSD group, HSD group had increased trabecular separation (Tb. Sp.) (p < 0.01) and trabecular pattern factor (Tb. Pf.) (p < 0.01) (Table 1). Together our data clearly demonstrates that HSD intake significantly enhances bone loss by impairing LV-5 vertebrae bone microarchitecture of mice. On the contrary LSD significantly inhibits deterioration of bone microarchitecture in LV5 vertebrae.

**Table 1:** Bone histomorphometric parameters of trabecular and cortical bones.

Bone parameters	Normal	LSD	HSD
LV5			
BV/TV (%)	25.52 ± 0.05	28.65 ± 0.09	21.59 ± 0.84**
Tb. Th (mm)	1.23 ± 0.04	1.31 ± 0.16	1.12 ± 0.07**
Tb. No (mm^−1^)	3.87 ± 0.42	4.35 ± 0.04	3.41 ± 0.50**
Conn. Den (mm^−3^)	2.53 ± 0.05	3.76 ± 0.09	1.97 ± 0.02**
Tb. Sp. (mm)	0.87 ± 0.08	0.42 ± 0.04	1.43 ± 0.34**
Cl. Po (%)	2.54 ± 0.37	2.12 ± 0.16	5.76 ± 0.46**
Femur Trabecular			
BV/TV (%)	34.21 ± 1.32	38.25 ± 1.24	25.43 ± 1.51**
Tb. Th (mm)	1.53 ± 0.24	1.78± 0.26	1.12 ± 0.18**
Tb. No (mm^−1^)	4.67 ± 0.21	5.21 ± 0.33	3.97 ± 0.18**
Conn. Den (mm^−3^)	3.87 ± 0.68	4.32 ± 0.84	3.61 ± 0.06*
Tb. Sp. (mm)	0.89 ± 0.01	0.54 ± 0.06	0.98 ± 0.08**
Cl. Po (%)	0.87 ± 0.09	0.62 ± 0.03	1.85 ± 0.13**
Tibia Trabecular			
BV/TV (%)	31.33 ± 0.75	36.22 ± 0.45	27.43 ± 0.34**
Tb. Th (mm)	1.21 ± 0.09	1.40 ± 0.08	1.06 ± 0.04*
Tb. No (mm^−1^)	3.21 ± 0.47	3.73 ± 0.51	3.01 ± 0.15*
Conn. Den (mm^−3^)	4.30 ± 0.47	4.73 ± 0.56	3.76 ± 0.48**
Tb. Sp. (mm)	1.37 ± 0.23	0.87 ± 0.07	1.46 ± 0.12**
Cl. Po (%)	0.85 ± 0.04	0.59 ± 0.02	2.12 ± 0.25**
Femur Cortical			
Tt. Ar (mm^2^)	2.56 ± 0.02	2.88 ± 0.10	1.85 ± 0.11**
T. Pm (mm)	0.57 ± 0.06	0.77 ± 0.07	0.45 ± 0.04**
Ct. Ar (mm^2^)	0.67 ± 0.01	0.89 ± 0.08	0.42 ± 0.06*
B. Pm (mm)	1.02 ± 0.07	1.32 ± 0.10	0.86 ± 0.09**
Ct. Th (mm)	0.76 ± 0.01	0.94 ± 0.06	0.54 ± 0.07**
MMI (mm^4^)	0.42 ± 0.04	0.61 ± 0.05	0.22 ± 0.07**
Tibia Cortical			
Tt. Ar (mm^2^)	1.88 ± 0.06	2.32 ± 0.91	1.58 ± 0.0.98**
T. Pm (mm)	0.63± 0.08	0.88 ± 0.07	0.48 ± 0.04*
Ct. Ar (mm^2^)	0.49 ± 0.02	0.69 ± 0.07	0.31 ± 0.03*
B. Pm (mm)	0.85 ± 0.17	1.01 ± 0.14	0.73 ± 0.12*
Ct. Th (mm)	0.56 ± 0.11	0.85 ± 0.19	0.47 ± 0.11**
MMI (mm^4^)	0.54 ± 0.07	0.71 ± 0.10	0.32 ± 0.06*

High dietary salt intake impairs both trabecular and cortical bone microarchitecture. Histomorphometric parameters of trabecular and cortical bones of LV5, femur and tibia of normal, low salt diet group (LSD) and high salt diet group (HSD). Bone volume/tissue volume ratio (BV/TV); Trabecular thickness (Tb. Th); Trabecular number (Tb. No.); Connectivity density (Conn. Den); Trabecular separation (Tb. Sep.); Trabecular pattern factor (Tb. Pf.); Total cross-sectional area (Tt. Ar.); Total cross-sectional perimeter (T. Pm); Cortical bone area (Ct. Ar); Bone perimeter (B. Pm); Average cortical thickness (Ct. Th); Polar moment of inertia (MMI). The results were evaluated by using ANOVA with subsequent comparisons by Student t test for paired or nonpaired data, as appropriate. Inner values are expressed as mean ± SEM (n = 10) and similar results were obtained in three independent experiments. Statistical significance was defined as p ≤ 0.05 (*p ≤ 0.05, **p ≤ 0.01, ***p ≤ 0.001) with respect to low salt diet group.

### High dietary salt intake impairs both femoral and tibial bone microarchitecture in mice

Since bone micro-architecture consists of both the trabecular and cortical parameters, we were interested in quantitating both separately in femur and tibia respectively. In gross observation by 3D-μCT of femur/tibial bones collected for µCT analysis, significant deterioration in the femur trabecular architecture was readily observed in HSD group when compared with LSD group (Fig. 2). The HSD group has decreased BV/TV (p < 0.01), Tb. Th (p < 0.01), Tb. N (p < 0.01), Conn. Den (p < 0.05) (Table 1); but increased Tb. Sp (p < 0.01), Tb. Pf (p < 0.01) (Table 1) in comparison to LSD intake group. Similar results were also observed in femur cortical bones (Table 1) with significant decrease in the cortical micro-architecture in HSD intake group as compared with LSD intake group. Additionally our data of μCT analysis of tibia trabecular micro-architecture of HSD group showed a clear and significant deterioration even in trabecular bone of tibia as compared with LSD intake group (Fig. 2). The HSD intake group has reduced BV/TV (p < 0.01), Tb. Th (p < 0.05), Tb. N (p < 0.05) and Conn. Den (p < 0.01); but increased Tb. Sp (p < 0.01) and Tb. Pf (p < 0.01) (Table 1) in comparison to LSD intake group. Similar results were also observed in tibial cortical bones (Table 1) with significant deterioration in cortical microarchitecture of bones in HSD intake group as compared with LSD group. Altogether, these results further support and strengthen our observations that intake of HSD in mice impairs both femoral and tibial bone micro-architecture. Notably, we also observed the significant role of LSD in maintaining as well as protecting both femoral and tibial bone microarchitecture in mice.

### High dietary salt intake decreases both mineral density and heterogeneity of bones

Bone mineral density (BMD) defines the quantity of mineralized tissue present (size and density) in bones and is usually considered as an important parameter for determining the tendency of bones to undergo fracture. Thus, in line with these findings, we next determined the effect of HSD intake on BMD for both cortical and trabecular bones. Interestingly, both trabecular and cortical bones from femur/tibia/LV5 illustrated a significant decrease in their respective BMDs in HSD group (Table 2). The BMDs of HSD intake group was significantly reduced as compared to that of LSD intake group in LV-5-trabecular (p < 0.01), femur trabecular (p < 0.01), tibia trabecular (p < 0.01), tibia cortical (p < 0.05) and femur cortical (p < 0.01) (Table 2). Moving ahead, we next investigated the effect of HSD intake on heterogeneity of bone content with the help of FTIR analysis. Mice were sacrificed at the end of experiment and femur cortical bones were collected for FTIR analysis of heterogeneity. Importantly, we observed that HSD induces significant reduction in heterogeneity of bones (determined by m/m, XST and c/p ratios). We observed that intake of HSD in mice significantly increased the m/m ratio (p < 0.05), XST (p < 0.05) and c/p ratio (p < 0.05) in femur cortical with respect to LSD intake group (Table 3). In summary, our data for the first time demonstrates that HSD intake alters natural physiology (loss of heterogeneity) of bones in mice, thereby making them susceptible to enhanced fracture risk, unlike LSD intake which significantly maintains this heterogeneity thereby leading to enhanced BMD.

**Table 2:** High dietary salt intake decreases mineral density of bones.

	LV5 (gm HA/cm^3^)	Femur Trabecular (gm HA/cm^3^)	Tibia Trabecular (gm HA/cm^3^)	Femur Cortical (gm HA/cm^3^)	Tibia Cortical (gm HA/cm^3^)
BMD of trabecular and cortical bones					
Normal	3.21 ± 0.8	4.57 ± 0.08	4.21 ± 0.38	1.32 ± 0.02	1.10 ± 0.12
LSD	3.52 ± 0.10	5.02 ± 0.12	4.89 ± 0.09	1.64 ± 0.26	1.52 ± 0.33
HSD	2.20 ± 0.08**	2.21 ± 0.44**	1.89 ± 0.28**	0.92 ± 0.02*	0.91 ± 0.10**

Tabular representation of BMD of trabecular (LV5, femur and tibia) and cortical (femur and tibia only) bones of normal, low salt diet (LSD) and high salt diet groups (HSD). The results were evaluated by using ANOVA with subsequent comparisons by Student t test for paired or nonpaired data, as appropriate. Analysis was performed using Sigma plot software (Systat Software, Inc., Germany). Values are reported as mean ± SEM (n = 10) and similar results were obtained in three independent experiments. Statistical significance was defined as p ≤ 0.05 (*p ≤ 0.05, **p ≤ 0.01) with respect to low salt diet group.

**Table 3:** High dietary salt intake decreases heterogenity of bones.

	Mineral/ matrix ratio (m/m)	Crystallinity (XST)	Carbonate/ phosphate ratio (c/p)
Compositional changes in cortical bones			
Normal	2.77 ± 0.2	1.12 ± 0.07	1.31 ± 0.11
LSD	2.55 ± 0.05	1.01 ± 0.06	1.18 ± 0.03
HSD	2.98 ± 0.5*	1.15 ± 0.12*	1.34 ± 0.02*

Tabular representation of compositional changes in bones as detected by FTIR for bone mineral/ organic matrix ratio (m/m), crystallininty (XST) and carbonate to phosphate ratio (c/p) of normal, low salt diet (LSD) and high salt diet groups (HSD). The results were evaluated by using ANOVA with subsequent comparisons by Student t test for paired or nonpaired data, as appropriate. Analysis was performed using Sigma plot software (Systat Software, Inc., Germany). Values are reported as mean ± SEM (n = 10) and similar results were obtained in three independent experiments. Statistical significance was defined as p ≤ 0.05 (*p ≤ 0.05, **p ≤ 0.01) with respect to low salt diet group.

### High dietary salt intake enhances bone loss by modulating Treg-Th17 cell balance

**Figure 3:**
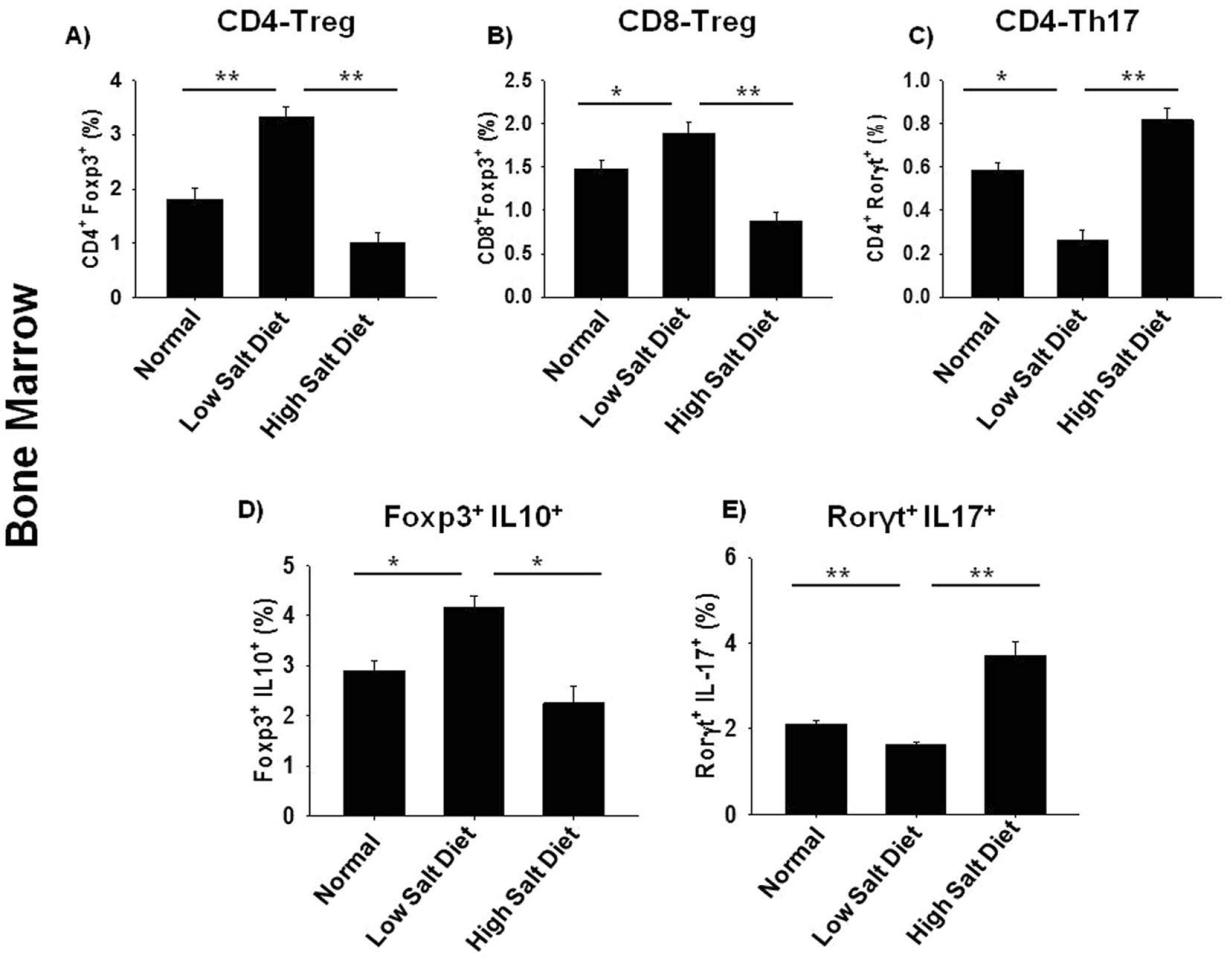
High dietary salt intake enhances bone loss by modulating Th17-Treg cell balance. Cells from bone marrow (BM) of normal, low salt diet (LSD) and high salt diet mice groups (HSD) were isolated at the end of experiment, labelled and analysed by flow cytometry for percentage of Foxp3^+^ and Rorγt^+^, IL-10^+^Foxp3^+^ and Rorγt^+^IL-17^+^ cells. Gating was first performed for CD4^+^ and later analysed for the expression of CD4^+^Foxp3^+^ (Tregs), CD4^+^Rorγt^+^ (Th17), CD4^+^IL-10^+^ (Tregs) and CD4^+^IL-17^+^ (Th17). (**A**) Average percentage of CD4^+^Foxp3^+^T cells (**B**) Average percentage of CD8^+^Foxp3^+^T cells (**C**) Average percentage of CD4^+^Rorγt^+^T cells. (**D**) Average percentage of Foxp3^+^IL-10^+^ (Treg). (**E**) Average percentage of Rorγt^+^ IL-17^+^ (Th17). The results were evaluated by using ANOVA with subsequent comparisons by Student t test for paired or nonpaired data, as appropriate. Analysis was performed using Sigma plot software (Systat Software, Inc., Germany). Values are reported as mean ± SEM (n = 10) and similar results were obtained in three independent experiments. Statistical significance was defined as p ≤ 0.05 (*p ≤ 0.05, **p ≤ 0.01, ***p ≤ 0.001) with respect to low salt diet group.

The role of Treg and Th17 cells in modulating bone health is now well established. Thus, to delineate the role of Treg and Th17 cells in HSD induced bone loss, we carried out flow cytometric analysis of bone marrow for both Treg and Th17 cells (Supplementary Fig. S4 and S5). Mice were sacrificed at completion of 45 days of HSD administration and total lymphocyte populations derived from bone marrow was analysed for CD4^+^Foxp3^+^ (CD4-Tregs), CD8^+^Foxp3^+^ (CD8-Tregs) and CD4^+^Rorγt^+^ (Th17) cells. As compared to LSD intake group, HSD group reported 2-fold decrease in CD4^+^Foxp3^+^Treg cells in bone marrow (3.75 ± 0.41% in low salt group to 1.2 ± 0.12% in high salt) (p < 0.01) (Fig. 3A). Interestingly, HSD intake also led to 2-fold decrease in CD8^+^Foxp3^+^ Treg cells in bone marrow (1.81 ± 0.02% in low salt group to 0.61 ± 0.01% in high salt) (p < 0.05) (Fig. 3B) as compared to LSD intake group. Next, we determined the effect of HSD intake on Th17 cells, notably in HSD intake group we observed more than three-fold increase in percentage of CD4^+^Rorγt^+^Th17 cells in bone marrow (0.41 ± 0.003% in LSD group to 0.78 ± 0.03% in HSD group) (p < 0.05) (Fig. 3C) with respect to LSD group.

Our next aim was to investigate the functionality of Treg and Th17 cells as determined by their cytokine production of IL-10 and IL-17 cytokines respectively in bone marrow (prime site of osteoclastogenesis). To explore the source of IL-10 and IL-17 secretion, total lymphocytes from bone marrow were activated with PMA (50 ng/ml) and ionomycin (500 ng/ml) for 5–6 h before analysis. Gating was performed first for total CD4^+^ T cells and later analysed for the expression of Foxp3^+^IL-10^+^ (Treg) cells and Rorγt^+^IL-17^+^ (Th17) cells. We observed that HSD intake in mice led to more than 2-fold decrease in Foxp3^+^IL-10^+^ cells in bone marrow (4.21 ± 0.32% in LSD group to 2.11 ± 0.41% in HSD group) (p < 0.05) (Fig. 3D) along with 3-fold increase in Rorγt^+^IL-17^+^ cells in bone marrow (1.78 ± 0.18% in LSD group to 3.87 ± 0.32% in HSD group) (p < 0.01) (Fig. 3E). Collectively, these results clearly demonstrate that HSD intake enhances production of IL-17 from Th17 cells along with simultaneously inhibiting production of IL-10 from Tregs. Interestingly, in LSD we observed a significant increase in the percentage of Treg cells along with simultaneous decrease in the percentage of Th17 cells. Altogether, our results for the first time establish the role of HSD intake in augmenting Treg-Th17 cell axis by enhancing Th17 cells and decreasing the percentage of Treg cells *in vivo* leading to enhanced bone loss in HSD group.

### High dietary salt intake skews expression of osteoclastogenic factors

**Figure 4:**
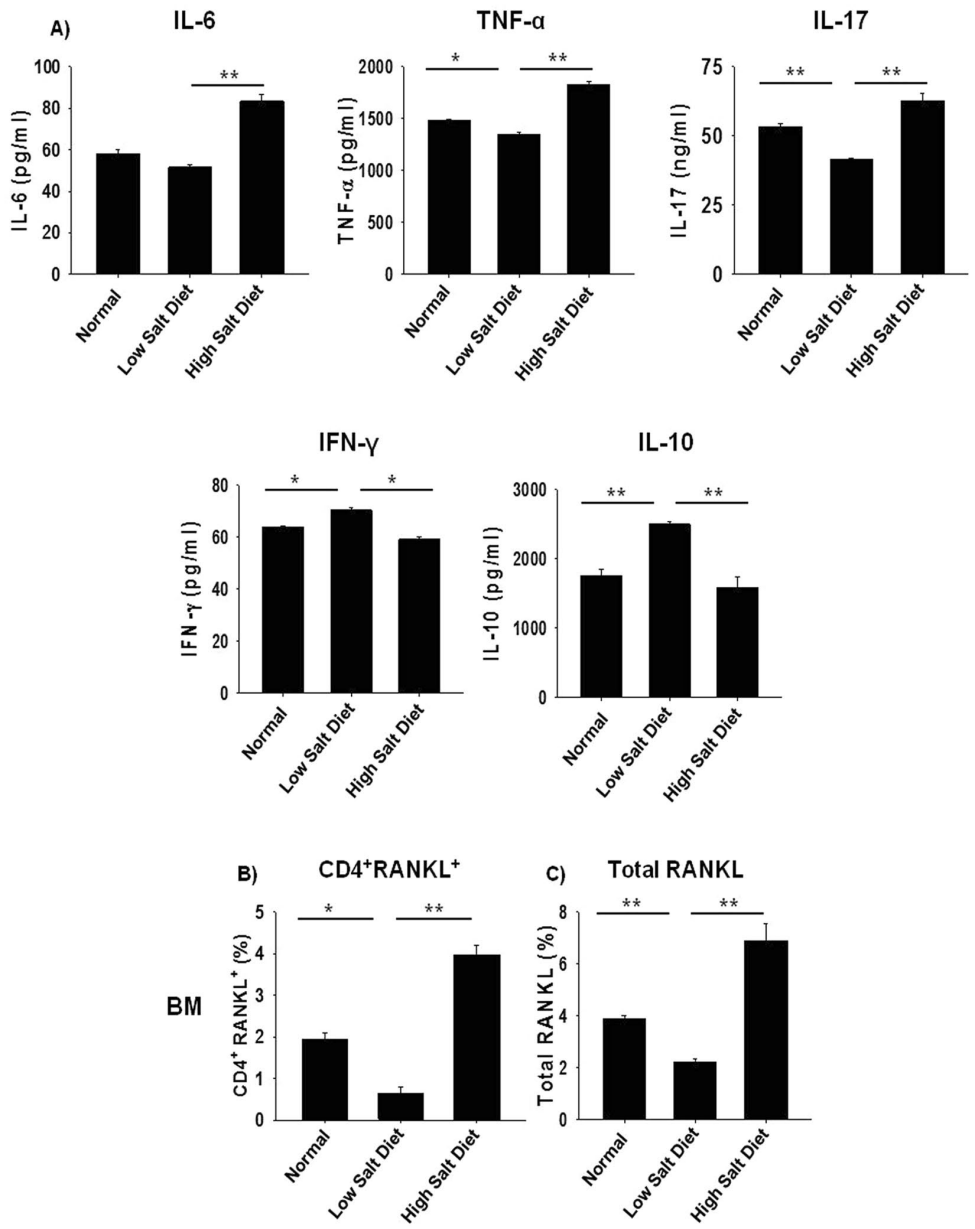
High dietary salt intake skews expression of osteoclastogenic factors. (**A**) Serum samples of mice were analyzed for secretion of various proinflammatory and anti-inflammatory cytokines by CBA (**B**) Average percentage (%) of CD4^+^RANKL^+^T cells in bone marrow (BM). (**C**) Average percentage (%) of total RANKL in bone marrow (BM). The results were evaluated by using ANOVA with subsequent comparisons by Student t test for paired or nonpaired data, as appropriate. Analysis was performed using Sigma plot software (Systat Software, Inc., Germany). Values are reported as mean ± SEM (n = 10) and similar results were obtained in three independent experiments. Statistical significance was defined as p ≤ 0.05 (*p ≤ 0.05, **p ≤ 0.01, ***p ≤ 0.001) with respect to low salt diet group.

**Figure 5:**
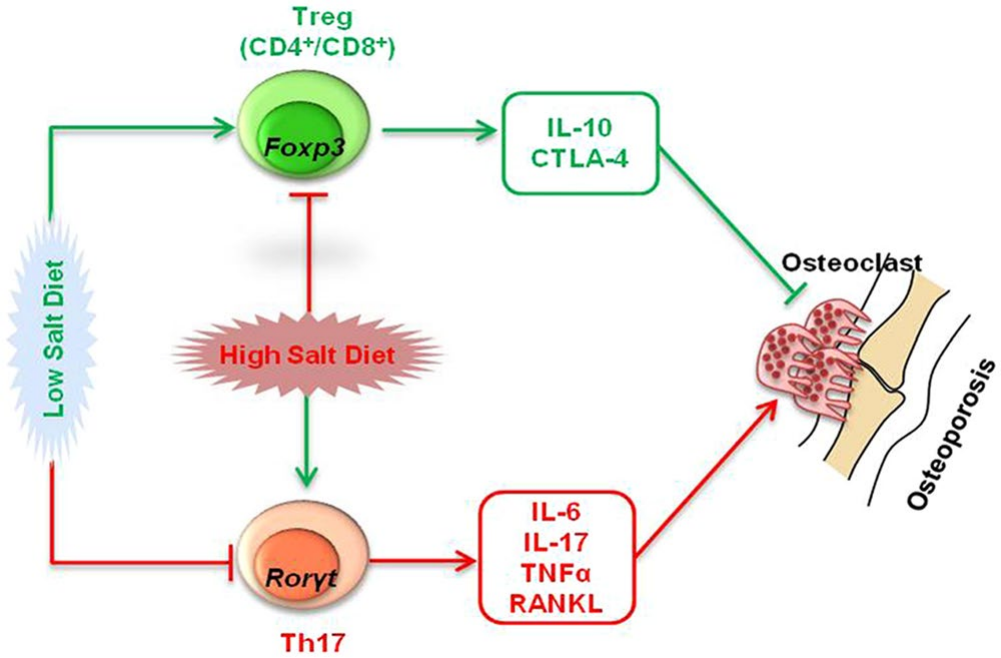
Summary of our results. High dietary salt intake induces bone loss by modulating Treg-Th17 balance *in vivo*.

To determine the effect of HSD on various proinflammatory cytokines, mice were sacrificed at the end of the experiment and blood serum cytokine levels was analysed by cytometric bead array (CBA). It was observed that HSD intake significantly increases levels of osteoclastogenic cytokines viz. IL-6 (p < 0.01), TNF-α (p < 0.01) and IL-17A (p < 0.01) in comparison to LSD intake group, which significantly inhibits the same (Fig. 4A). Interestingly, HSD intake significantly decreased the levels of IFN-γ (p < 0.05) and IL-10 (p < 0.01) which are known anti-osteoclastogenic cytokines^32^. Since RANKL has been reported as the prime factor for inducing osteoclastogenesis and is known to be expressed by activated T cells (including Th17 cells)^32^. Thus, it’s important to determine the amount and source of RANKL in our system. In line with this we next examined the amount of total RANKL produced by all sources, along with RANKL secreted by T cells alone. Excitingly, we observed that intake of HSD in mice results in a two-fold increase in RANKL secreting CD4^+^T cells in bone marrow (0.81 ± 0.05% in LSD group to 4.01 ± 0.32% in HSD group) (p < 0.01) with respect to LSD intake group (Fig. 4B). Simultaneously, the total RANKL expression was also significantly increased in HSD group in bone marrow (2.01 ± 0.27% in LSD group to 7.11 ± 0.94% in HSD group) (p <  0.01) with respect to LSD intake group (Fig. 4C). Importantly, in LSD group we observed a significant reduction in the production of proinflammatory factors/cytokines along with an increase in the production of anti-inflammatory factors thereby leading to enhanced bone health observed in LSD group. Taken together, our results confirm that HSD intake enhances production of proinflammatory factors/cytokines and inhibits secretion of anti-inflammatory factors, thereby enhancing bone loss in mice (Fig. 5).

## Discussion

The incidence of various inflammatory diseases in the society at large is increasing rapidly, suggesting the influence of environmental factors such as diet as an important trigger for a number of diseases^4,5^. The “Western diet,” high in saturated fatty acids and salt has long been postulated as one of the potential environmental cause for the increasing incidence of inflammatory diseases in developed countries where processed and fast food consumption is more common^33^. HSD has been implicated in the development of hypertension, chronic inflammation, cancer and autoimmune diseases^34^. Dietary salt (sodium chloride-NaCl) intake in our foods varies vastly, ranging from less than 1 g/day in homemade food to more than 20 g/day in the western world^9^. It is to be noted that sodium content of processed foods and ‘fast food’ preferentially consumed now a days can be more than 100 times higher in comparison to similar homemade meals^9^. As already observed, dietary salt intake has been main culprit in the development of various diseases such as cardiovascular disease and stroke^35,36^. High-salt diets have also been reported to result in interstitial hypertonic Na^+^ accumulation in the skin and muscle thereby activating tissue-resident macrophages to secrete endothelial growth factor-C, which in turn increases interstitial hypertonic volume retention and blood pressure^37^. Notably, these complications were not reported in diets with low salt or moderate salt content, where the normal physiology and health was least affected^35–37^. In fact, low salt intake or moderate salt diet was beneficial in patients suffering from cardiovascular diseases^37^. Within tissue inflammatory sites, elevated sodium also increases the development of proinflammatory M1 macrophages^29^. The pathogenesis of several of these diseases is characterized by deregulation of Treg-Th17 cell axis and induction of various pro and anti-inflammatory cytokines like TNF-α, IL-6, IFNγ and IL-10. Recent reports have shown that the differentiation of Th17 cells is sensitive to changes in local microenvironments, such as salt (NaCl)^38^. Moreover, increased sodium promotes the development of IL-17 producing CD4^+^ T cells (Th17 cells), which are critical effector cells in response to extracellular bacterial infections and are also important mediators of autoimmune inflammatory diseases^39^. This increased Th17 response occurs via sodium enhancement of serum/glucocorticoid regulated kinase 1 (SGK1) activation^38^. Thus, in addition to increasing blood pressure and the risk of stroke, increased salt intake can undermine the course and balance of the immune response by promoting the development of macrophages and T cells with proinflammatory functions^30,38^.

Recently, several studies have depicted the role of dietary salt intake on bone health. Devine *et al*. studied the relationship of salt intake and bone mineral density by linking dietary calcium intake and urine sodium excretion^40^. In another study, it was reported that reduction of sodium intake leads to improved bone health^41^. Similarly, Jones *et al*. reported that intake of HSD in men and women are associated with higher bone resorption markers, thereby increasing the risk of osteoporosis^42,43^. Since Th17 and Treg have established role in bone health along with the fact that high salt induces Th17^39^ and impairs Tregs^30^, we were interested in elucidating the role of HSD on bone health via its effect on Treg-Th17 cell axis. Also, no study till date had ever unravelled the role of Th17-Treg cells in high dietary salt induced bone loss. Our present study thus for the first time demonstrates that higher intake of dietary salt (sodium chloride) enhances bone loss in male mice. To elucidate the mechanism of HSD intake in regulating bone health we started to investigate its effect on host physiology, particularly the osteoimmune system. SEM and AFM analysis of bone samples confirmed that HSD intake enhances bone loss in mice. µCT of bones further reaffirmed our results, as significant bone loss was observed in LV-5, femur and tibial bones of HSD mice group with respect to LSD and normal groups. Interestingly, LSD resulted in enhanced bone mass, along with improved bone microarchitecture in LV-5, femur and tibial bones, as confirmed by SEM, AFM and µCT analysis. These results clearly validate the effect of both HSD and LSD on bone health.

Bone is a dynamic and heterogeneous tissue consisting of different constituents viz. mineral phase (hydroxyapatite), organic phase (∼90% collagen type I, ∼5% noncollagenous proteins, ∼2% lipids) and water^44,45^. With the advancement of age, gender and diet their relative proportions in the bone vary significantly. The bone has heterogeneous composition due to its constant remodelling and mineralization cycles. Loss of material heterogeneity is associated with an increase in brittleness thereby increasing the risk of fracture^44^. Thus, the physiological composition of the bone in the healthy individual must be maintained for proper functioning of bones. Since loss of heterogeneity in bones has been associated with severe fractures and osteoporosis^44^, it thus makes sense to study the effect of HSD intake on bone heterogeneity parameters viz. crystallinity-XST (high XST ratio = enhanced bone loss); mineral to organic matter ratio-m/m (high m/m ratio = enhanced bone loss); and carbonate content-c/p (high c/p ratio = enhanced bone loss)^44,45^ along with BMD. Importantly, our results clearly indicate significant decrease in both the mineral density and heterogeneity of bones (as increase in m/m, XST and c/p ratios are related with enhanced bone loss in HSD mice group with respect to LSD mice group). Interestingly, the heterogeneity in case of LSD group mice was observed to be significant enhanced. These data clearly highlight the key role of HSD in enhancing bone loss, along with compromising on part of bone heterogeneity. Thus, our data for the first time demonstrate the ill effects of HSD intake on bone health leading to enhanced bone loss with simultaneous decreased heterogeneity of bones, an important factor for determining bone fragility and fracture risk in the long run^44^.

Several earlier studies have tried to link and establish the role of HSD with either immune system or bone health^34,39^. Treg cells (both CD4^+^ and CD8^+^) have long been associated with bone protective functions^46,47^, whereas Th17 has now been associated with inflammatory bone loss in RA, osteoporosis etc.^46^. It has been already reported that high salt intake enhances Th17 cells along with simultaneously impairing Treg cell functions^38,45^. Also, IL-17 plays an important role in osteoclast formation via its effect on NF-κB and RANKL secretion^48^. In line with these findings, we report for the first time that HSD in male mice significantly increases the population of Th17 cells and decreases Tregs (both CD4^+^ and CD8^+^ Treg) populations in bone marrow, a primary site of osteoclastogenesis. On the contrary in case of LSD mice there was observed a significant increase in the population of Treg cells along with simultaneous decrease in Th17 cell population. Taken together these results clearly demonstrate that HSD impairs bone health by enhancing osteoclastogenic Th17 cells and inhibiting anti-osteoclastogenic Treg cells.

The role of pro-inflammatory cytokines in the pathogenesis of inflammatory diseases is well established. Also, various studies have mentioned the negative effect of pro-inflammatory cytokines on bone health leading to several inflammatory conditions of bones such as osteoporosis and osteoarthritis^46^. Bone loss is regarded as an inflammatory condition because of the causal role of the immune system^49,50^, particularly due to the presence of increased levels of proinflammatory cytokines (IL-6, TNF-α, IL-17 and RANKL)^50–53^ along with decreased levels of anti-inflammatory factors (IL-10 and IFN-γ)^54,55^ resulting in enhanced bone loss. It is now well established that IL-10, secreted from Treg cells possess anti-inflammatory properties and is responsible for their suppressive functioning during inflammation^56^. On the other hand, Th17 cells produce IL-17, which is responsible for enhanced bone loss^57,58^. In our present study, we too report that HSD intake enhances bone loss in mice by stimulating production of proinflammatory cytokines (IL-6, IL-17 and TNF-α), along with decreasing production of anti-inflammatory cytokines (IFN-γ and IL-10). In addition, significant increase in the production of RANKL (an important mediator of osteoclastogenesis) from both T cells and non-T cell sources was observed in HSD intake group with respect to LSD intake mice group. Together our data clearly reveal and establish the key role of HSD in elevating the production of proinflammatory cytokines and simultaneously decreasing the production of anti-osteoclastogenic factors, thereby enhancing bone loss. It is worth mentioning here that LSD on the contrary to HSD results in a significant decrease in the production of proinflammatory cytokines (IL-6, IL-17 and TNF-α), along with enhanced production of anti-osteoclastogenic cytokines (IFN-γ and IL-10). Thus, our results not only highlight the adverse effects of HSD on bone health, they also point out the important protective role played by LSD in maintaining normal host physiology with respect to various inflammatory immune responses. Collectively, our results for the first time establish a strong correlation between HSD intake and bone loss via modulation of Th17-Treg cell balance *in vivo*. These results once again highlight the importance of dietary salt (particularly high salt) intake from various packed and processed food items which can ultimately increase the risk of various inflammatory diseases including bone health.

## Material and Methods

### Animals

Thirty male mice (BALB/c) of 8–10 weeks with an average body weight (mean ± standard error of the mean) of 27 g ±  2 g were selected. The high salt diet (HSD) group mice received diet containing 4% NaCl in chow plus 1% in drinking water. The low salt group mice were fed on diet having 0.4% NaCl in chow plus normal drinking water whereas normal mice were given a diet containing 0.8% NaCl in chow plus normal drinking water for 45 days. Mice were maintained under specific pathogen-free conditions and fed sterilized food and autoclaved water ad libitum. At the end of experiment (45 days), animals were sacrificed and bones, serum, organs collected for further analysis. All the procedures involving animals were conducted according to the requirements and with the approval of the Institutional Animal Ethics Committee of AIPS.

### Antibodies and Reagents

The following antibodies/kits were bought from BD Biosciences (USA): PerCP-Cy-5.5 Rat Anti-Mouse CD4-(RM4-5) (550954), APC-Rat-Anti-Mouse-CD8a-(53-6.7) (561093), PE-Rat-Anti-Mouse-CD45R/B220-(RA3-6B2) (553089) and mouse Cytometric Bead Array-(CBA) kit. The reagents Anti-Human/Mouse-Rorγt-PE-(AFKJS-9) (12-6988), Anti-Mouse/Rat-Foxp3-APC-(FJK-16s) (17-5773), Foxp3/Transcription factor staining buffer (0-5523-00), RBC lysis buffer (00-4300-54) were obtained from eBioscience (USA). Anti-Mouse-CD254-(TRANCE-RANKL) PE (IK22/5) (510005), Anti-Mouse-CD8-PE/Cy7 (53-6.7) (100721), Anti-Mouse-IFN-γ-FITC (XMG 1.2) (505805), Anti-Mouse-IL-10-FITC (JESS-16E3) (505005), Anti-Mouse-IL-17A-PE (TC11-8H10.1) (506903) were obtained from BioLegend (USA).

### Scanning Electron Microscopy (SEM)

For SEM analysis, the femur cortical samples were kept in 1% Triton for 48–72 hours and later transferred to 1xPBS solution till final analysis is done. Bone slices were made and dried under incandescent bulb before SEM analysis and scanned in NOVA NANOSEM 450 microscope equipped with a tungsten filament gun operating at WD 10.6 mm and 20 kV. SEM images were digitally photographed at low (15x), intermediate (1000x), and high magnifications (10,000x) to picture the best cortical structure. Images were later processed and analyzed using Adobe Photoshop 7.0.

### Atomic Force Microscopy (AFM)

Femur cortical bone samples were dried in dust free environment with 60 W lamps for 6 hours followed by high vacuum drying and subsequently analysed by Atomic Force Microscope (INNOVA, ICON, Bruker), which operates under the Acoustic AC mode (AAC or Tapping mode). This was assisted with cantilever (NSC 12(c) from MikroMasch, Silicon Nitride Tip) by NanoDrive^TM^ version 8 software at a force constant of 0.6 N/m, having resonant frequency at 94–136 kHz. The images were taken in air at room temperature at the scan speed of 1.5–2.2 lines/s. The data analysis was done using of Nanoscope analysis software. The data was further analyzed using MATLAB software (Mathwork, USA).

### Micro computed Tomography (μ-CT)

μ-CT of the femur/tibia (trabecular/ cortical) and lumbar vertebrae-V (trabecular) was performed using SkyScan 1076 scanner (SkyScan). Scanning was done at 70 kV, 100 mA using a 0.5-mm aluminum filter and exposure set to 590 ms. In total, 1800 projections were collected at a resolution of 6.93 μm/pixel. For purpose of carrying out reconstruction process NRecon software was used. Bone length was determined from the rendered 3D images in CTAn software. The distance from trochanter to edge of femoral condyles defined total femoral length. Tibial length calculated as distance from medial condyle to medial malleolus. Manual segmentation of 2D Slice of sagittal images was done to isolate growth plates from the surrounding bone tissue in micro-CT images followed by reconstruction to render 3D images. It was from these images that growth plate height was measured using data viewer software. For trabecular region analysis, ROI was drawn at a total of 100 slices in secondary spongiosa at a distance of 1.5 mm from distal border of growth plates excluding the parts of cortical bone and primary spongiosa. *In vivo* measurement of LV5 trabecular was done by encompassing 50 continuous slices which start from the beginning of trabecular bone within spinal body^58,59^. The CTAn software was used to analyze cortical bone by taking into consideration 350 consecutive image slides discarded from growth plate to leave out all trabecular regions, out of these 200 continuous images were selected as such. Various bone histomorphometric trabecular parameters (3D) and cortical parameters (2D) were analyzed by already established protocols^60,61^. For determination of BMD of LV5, femur and tibia μ-CT scans were utilized as determined from VOI made for trabecular and cortical region. For BMD calibration, 2-mm-diameter hydroxyapatite phantom rods with known BMD (0.25 and 0.75 g/cm^3^) were used. For each analysis, the estimated BMD was determined based on linear correlation between the µCT attenuation coefficient and BMD^61^.

### FTIR

The FTIR analysis of femur cortical bones was done by using 8400S-Fourier Transform Infrared Spectrophotometer (SHIMADZU), with a resolution 4 cm^−1^; scan speed 2.5 kHz, and 128 scans co-addition, in the KBr pellet form. Savitzky-Golay algorithm was used to eliminate the noise for obtaining smooth spectra of samples. The samples were held in contact with a prism made of highly refractive material transmitting infrared rays which are made incident on the sample at angle that induces total reflection. Bones were kept in 1% Triton, washed after 24 hours and dried under 60 W lamps for 6 hours followed by high vacuum drying. Dry bones were made into powder form and mixed with KBr (potassium bromide) in 1:100 ratios and analysed for FTIR.

### Flow Cytometry

Briefly, T cells in single-cell suspensions isolated from bone marrow and spleen were stained with PerCP-Cy5.5-anti-CD4 and PE/Cy-7-anti CD8 or APC-anti-CD8. They were allowed to pellet down after centrifugation at 1800 rpm for 5 minutes followed by addition of 1 ml RBC lysis buffer. Cells were washed thoroughly with wash buffer and fixed-permeabilized with Foxp3 permeabilization buffer for 30 minutes on ice. Intracellular Foxp3, RANKL and Rorγt staining was performed using APC-conjugated anti-Foxp3, PE-conjugated anti-RANKL and PE-conjugated anti-Rorγt Abs in permeabilization buffer for 45 minutes on ice. Cells were washed thoroughly with permeabilization buffer and analyzed by flow cytometry.

Intracellular staining for IL-10, IL-17, IFN-γ production for Tregs and Th17 population was performed by stimulating cells for 5–6 hours with PMA (50 ng/ml) and ionomycin (500 ng/ml). After 1 hour, GolgiStop (1 μg/ml) was added in the cultures to block cytokine secretion. Cells were first surface stained with PerCP-Cy5.5-anti-CD4 and PE/Cy-7-anti CD8 antibodies for 30 minutes on ice. Cells were washed thoroughly with wash buffer and further fixed-permeabilized with Foxp3 fixation-permeabilization buffer for 30 minutes on ice. Cells were washed thoroughly with Foxp3-permeabilization buffer and stained intracellularly with APC conjugated anti-Foxp3 and PE-conjugated anti-IFN-γ; FITC-conjugated anti-IL-10 and PE- conjugated IL-17A antibodies in permeabilization buffer for 30 minutes on ice^61^. Cells were washed thoroughly with Foxp3-permeabilization buffer and analyzed by flow cytometry on BD FACS ARIA III (BD Biosciences), and the data was analyzed using FloJo (TreeStar, USA).

### Cytometric Bead Array (CBA)

Levels of IL-6, IL-10, IL-17A, IFN-γ and TNF-α in blood serum were assessed by fluorescent bead-based technology using CBA kit and all procedures were followed according to the manufacturer’s instruction (BD Biosciences, USA). Fluorescent signals were read and analysed on a FACS Aria III flow cytometer (BD Biosciences) with the help of BD FCAP Array software (BD Biosciences).

### Statistical analysis

The results were evaluated by using ANOVA with subsequent comparisons by Student t test for paired or nonpaired data, as appropriate. Analysis was performed using Sigma plot software (Systat Software, Inc., Germany). Values are reported as mean  ±  SEM (n=10). Statistical significance was defined as p ≤ 0.05 (*p ≤ 0.05, **p ≤ 0.01, ***p ≤ 0.001) with respect to low salt group.

## Electronic supplementary material

Supplementary Information

## References

[1] Laird, E. *et al*. Greater yogurt consumption is associated with increased bone mineral density and physical function in older adults. *Osteoporos. Int* 1–11, 10.1007/s00198-017-4049-5 (2017). 10.1007/s00198-017-4049-5 28462469

[2] Lindsay R (2001). Risk of new vertebral fracture in the year following a fracture. JAMA.

[3] Ilich JZ, Kerstetter JE (2000). Nutrition in bone health revisited: a story beyond calcium. J Am Coll Nutr.

[4] Sigaux, J. *et al*. Salt, Inflammatory Joint Disease, and Autoimmunity. *Joint Bone Spine*, 10.1016/j.jbspin.2017.06.003 (2017). 10.1016/j.jbspin.2017.06.003 28652101

[5] de Carvalho JF (2009). The mosaic of autoimmunity: the role of environmental factors. Front Biosci (Elite Ed).

[6] Heaney RP (2000). Calcium, dairy products and osteoporosis. J Am Coll Nutr.

[7] Cohen AJ, Roe F (2000). J. C. Review of risk factors for osteoporosis with particular reference to a possible aetiological role of dietary salt. Food Chem Toxicol.

[8] Teucher B, Fairweather-Tait S (2003). Dietary sodium as a risk factor for osteoporosis. Where is the evidence?. Proc Nutr Soc.

[9] Brown IJ (2009). Salt intakes around the world: implications for public health. Int J Epidemiol.

[10] Guideline: Sodium Intake for Adults and Children. Geneva: World Health Organization 2012. http://www.ncbi.nlm.nih.gov/books/NBK133309/ (accessed 21 Sep 2017). 23658998

[11] Appel LJ (2011). The importance of population-wide sodium reduction as a means to prevent cardiovascular disease and stroke: a call to action from the American Heart Association. Circulation.

[12] He FJ, MacGregor GA (2010). Reducing population salt intake worldwide: from evidence to implementation. Prog Cardiovasc Dis.

[13] Cook NR (2007). Long term effects of dietary sodium reduction on cardiovascular disease outcomes: observational follow-up of the trials of hypertension prevention (TOHP). BMJ.

[14] Cianciaruso B (1998). Salt intake and renal outcome in patients with progressive renal disease. Miner Electrolyte Metab.

[15] Perry IJ, Beevers DG (1992). Salt intake and stroke: a possible direct effect. J Hum Hypertens.

[16] Kupari M, Koskinen P, Virolainen J (1994). Correlates of left ventricular mass in a population sample aged 36 to 37 years. Focus on lifestyle and salt intake. Circulation.

[17] Nishida C (2004). The joint WHO/FAO expert consultation on diet, nutrition and the prevention of chronic diseases: process, product and policy implications. Public Health Nutr.

[18] Teucher B, Fairweather-Tait S (2003). Dietary sodium as a risk factor for osteoporosis: where is the evidence?. Proc Nutr Soc.

[19] Teucher B (2008). Sodium and bone health: impact of moderately high and low salt intakes on calcium metabolism in postmenopausal women. J Bone Miner Res.

[20] Dar HY (2017). Osteoimmunology: The Nexus between bone and immune system. Frontiers In Bioscience, Landmark.

[21] Polanczyk MJ (2004). Cutting Edge: Estrogen Drives Expansion of the CD4+CD25+ Regulatory T cell Compartment. J. Immunol.

[22] Sato KA (2006). Th17 functions as an osteoclastogenic helper T cell subset that links T cell activation and bone destruction. J Exp Med.

[23] Yuan FL (2010). Regulatory T cells as a potent target for controlling bone loss. Biochem. Bio phys Res Commun.

[24] van Amelsfort JM (2004). CD4+CD25+ regulatory T cells in rheumatoid arthritis: differences in the presence, phenotype, and function between peripheral blood and synovial fluid. Arthritis Rheum..

[25] Jörg S (2016). High salt drives Th17 responses in experimental autoimmune encephalomyelitis without impacting myeloid dendritic cells. Exp Neurol..

[26] Farez MF, Fiol MP, Gaitan MI, Quintana FJ, Correale J (2015). Sodium intake is associated with increased disease activity in multiple sclerosis. J Neurol Neurosurg Psychiatry.

[27] Sundstrom B, Johansson I, Rantapaa-Dahlqvist S (2015). Interaction between dietary sodium and smoking increases the risk for rheumatoid arthritis: results from a nested case-control study. Rheumatology (Oxford).

[28] Ip WK, Medzhitov R (2015). Macrophages monitor tissue osmolarity and induce inflammatory response through NLRP3 and NLRC4 inflammasome activation. Nat Commun..

[29] Jantsch J (2015). Cutaneous Na+ storage strengthens the antimicrobial barrier function of the skin and boosts macrophage-driven host defense. Cell Metab.

[30] Amanda L (2015). Sodium chloride inhibits the suppressive function of FOXP3^+^ regulatory T cells. J. Clin. Invest.

[31] Thurner PJ (2007). High-speed photography of compressed human trabecular bone correlates whitening to microscopic damage. Eng. Fract. Mech.

[32] Zhang K (2011). CD8+ T cells regulate bone tumor burden independent of osteoclast resorption. Cancer Res.

[33] US Department of Agriculture, 2015–2020 Dietary Guidelines for Americans. 8th. ed. Washington DC, http://health.gov/dietaryguidelines/2015/guidelines/ (2015).

[34] Binger KJ (2015). High salt reduces the activation of IL-4- and IL-13-stimulated macrophages. J. Clin. Invest.

[35] Savica V, Bellinghieri G, Kopple JD (2010). The effect of nutrition on blood pressure. Annu Rev Nutr.

[36] Bragulat E, de la Sierra A (2002). Salt intake, endothelial dysfunction, and salt-sensitive hypertension. J Clin Hypertens (Greenwich).

[37] Braam B (2017). Understanding the Two Faces of Low-Salt Intake. Curr Hypertens Rep..

[38] Wu C (2013). Induction of pathogenic TH17 cells by inducible salt-sensing kinase SGK1. Nature.

[39] Kleinewietfeld M (2013). Sodium chloride drives autoimmune disease by the induction of pathogenic TH17 cells. Nature.

[40] Devine A (1995). A longitudinal study of the effect of sodium and calcium intakes on regional bone density in postmenopausal women. Am J Clin Nutr.

[41] Lin PH (2003). The DASH diet and sodium reduction improve markers of bone turnover and calcium metabolism in adults. J Nutr.

[42] Jones G (1997). A population-based study of the relationship between salt intake, bone resorption and bone mass. Eur J Clin Nutr.

[43] Frassetto LA, Morris RC, Sebastian A (2004). Dietary sodium as a determinant of bone resorption rate and bone mineral density in postmenopausal women. J Am Soc Neph.

[44] Boskey AL (2015). Bone composition: relationship to bone fragility and anti-osteoporotic drug effects. Bonekey Rep.

[45] Gadeleta SJ (2000). A physical, chemical, and mechanical study of lumbar vertebrae from normal ovariectomized, and nandrolone decanoate-treated cynomolgus monkeys (macaca fascicularis). Bone.

[46] D’Amelio P (2008). Estrogen deficiency increases osteoclastogenesis upregulating T cells activity: a key mechanism in osteoporosis. Bone.

[47] Pfeilschifter J (1989). Interleukin-1 and tumor necrosis factor stimulate the formation of human osteoclast like cells *in vitro*. J Bone Miner Res.

[48] Kotake S (1999). IL-17 in synovial fluids from patients with rheumatoid arthritis is a potent stimulator of osteoclastogenesis. J. Clin. Invest.

[49] Teitelbaum SL (2000). Bone résorption by osteoclasts. Science.

[50] Takayanagi H (2000). T-cell-mediated regulation of osteoclastogenesis by signaling cross-talk between RANKL and IFN-gamma. Nature.

[51] Devlin RD (1998). IL-6 mediates the effects of IL-1 or TNF, but not PTHrP or 1,25(OH)2D3, on osteoclast-like cell formation in normal human bone marrow cultures. J. Bone Miner. Res.

[52] Ma T (2004). Human interleukin-1-induced murine osteoclastogenesis is dependent on RANKL, but independent of TNF-α. Cytokine.

[53] Lee ZH (2002). IL-1α stimulation of osteoclast survival through the PI 3-kinase/Akt and ERK pathways. J. Biochem..

[54] Soysa NS (2012). Osteoclast formation and differentiation: an overview. J Med Dent Sci.

[55] Kelchtermans H, Billiau A, Matthys P (2008). How interferon-gamma keeps autoimmune diseases in check. Trends Immunol.

[56] Yago T (2009). IL-17 induces osteoclastogenesis from human monocytes alone in the absence of osteoblasts, which is potentially inhibited by anti-TNF-alpha antibody: a novel mechanism of osteoclastogenesis by IL-17. J Cell Biochem.

[57] De Benedetti F (2006). Impaired skeletal development in interleukin-6-transgenic mice: a model for the impact of chronic inflammation on the growing skeletal system. Arthritis Rheum..

[58] Li J-YJ (2016). Sex steroid deficiency-associated bone loss is microbiota dependent and prevented by probiotics. J. Clin. Invest.

[59] Dempster DW (2013). Standardized nomenclature, symbols, and units for bone histomorphometry: A 2012 update of the report of the ASBMR Histomorphometry Nomenclature Committee. J Bone Miner Res.

[60] Srivastava, K. *et al*. Greater skeletal gains in ovary intact rats at maturity are achieved by supplementing a standardized extract of Butea monosperma stem bark that confers better bone conserving effect following ovariectomy and concurrent treatment withdrawal, Evidence-Based Complement. *Altern. Med* 519387 (2013). 10.1155/2013/519387 PMC3655608 23710224

[61] Srivastava RK (2011). IL-3 attenuates collagen-induced arthritis by modulating the development of Foxp3+ regulatory T cells. J. Immunol.

