# Supplementary material for: High dietary salt intake correlates with modulated Th17-Treg cell balance resulting in enhanced bone loss and impaired bone-microarchitecture in male mice
Source: Sci Rep. 2018 Feb 6;8:2503. doi: 10.1038/s41598-018-20896-y (PMC5802842; doi:10.1038/s41598-018-20896-y)
Supplement: Supplementary file 1 — Supplementary Information [file 41598_2018_20896_MOESM1_ESM.pdf]

Supplementary Information

"High dietary salt intake correlates with modulated Th17-Treg cell balance resulting in enhanced bone loss and impaired bone-microarchitecture in male mice"

*Hamid Y. Dar[1], Anjali Singh[2], Prashant Shukla[3], Rajaneesh Anupam[2], Rajesh K. Mondal[4], Pradyumna K. Mishra[5] & Rupesh K. Srivastava[1†]**

[1]Department of Zoology, School of Biological Sciences, Dr. Harisingh Gour Central University, Sagar (MP)-470003, India
[2]Department of Biotechnology, School of Biological Sciences, Dr. Harisingh Gour Central University, Sagar (MP)-470003, India
[3]Department of Physics, School of Mathematical and Physical Sciences, Dr. Harisingh Gour Central University, Sagar (MP)-470003, India.
[4]Department of Microbiology, School of Biological Sciences, Dr. Harisingh Gour Central University, Sagar (MP)-470003, India.
[5]Department of Molecular Biology, National Institute for Research in Environmental Health, Bhopal (MP)- 462001, India
[†]Present address Department of Biotechnology, All India Institute of Medical Sciences (AIIMS), New Delhi-110029, India

*rupesh_srivastava13@yahoo.co.in

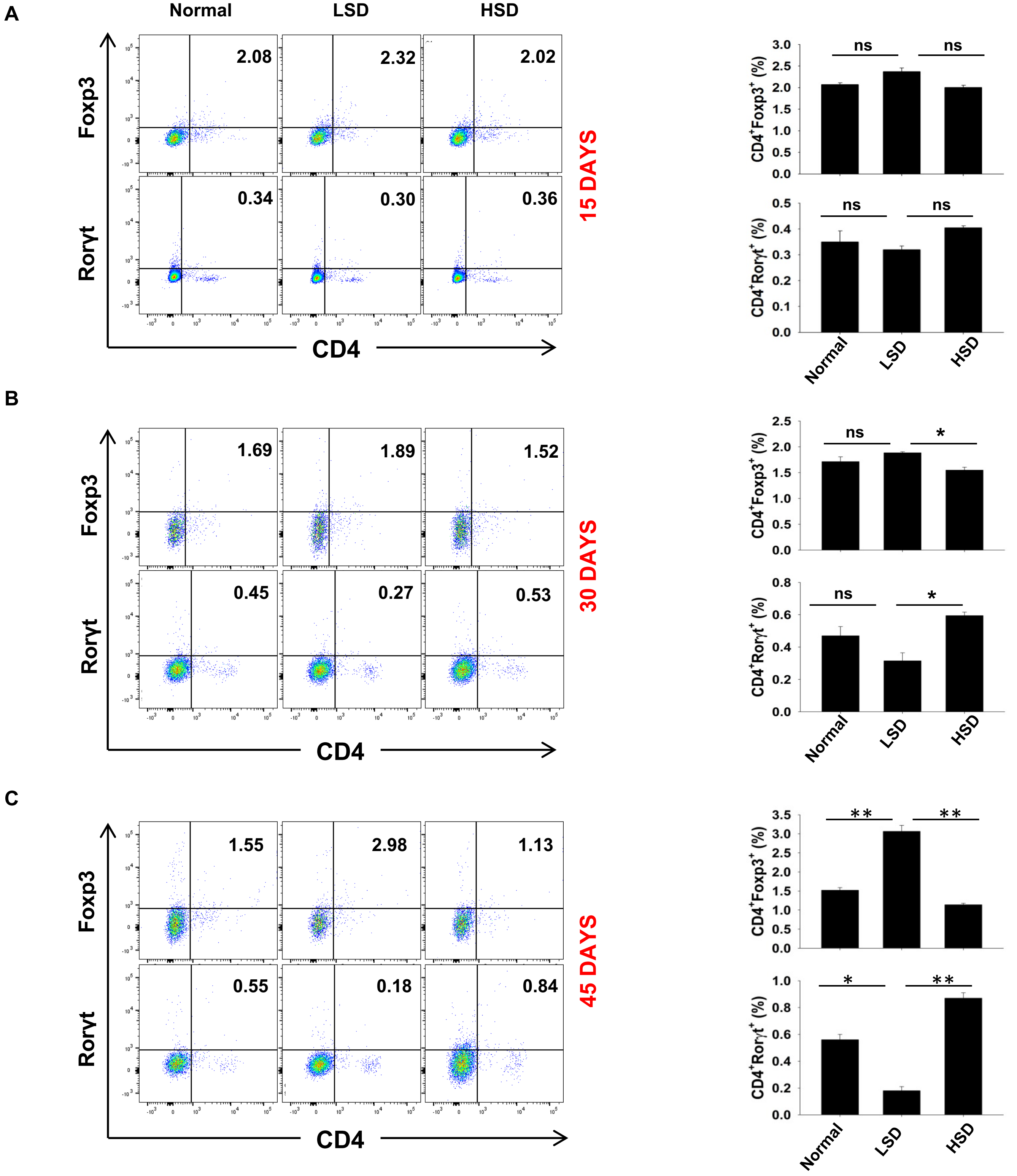

Supplementary Figure S1. Flow cytometry analysis of bone marrow cells at 15, 30 and 45 days intake of high dietary salt. Cells from bone marrow (BM) of normal, low salt diet (LSD) and high salt diet (HSD) mice groups were isolated at the end of respective time points (15, 30 and 45 days), labelled and analysed by flow cytometry for percentage of CD4$^+$ Foxp3$^+$ and CD4$^+$ Rorγt$^+$ T cells. Gating was first performed for CD4$^+$ T cells and later analysed for the expression of CD4$^+$Foxp3$^+$ (Tregs) and CD4$^+$Rorγt$^+$ (Th17) at 15, 30 and 45 days interval. **A).** Representative images of percentages of CD4$^+$Foxp3$^+$ and CD4$^+$Rorγt$^+$ T cells along with average percentage of CD4$^+$Foxp3$^+$ and CD4$^+$Rorγt$^+$ T cells at 15 days. **B).** Representative images of percentages of CD4$^+$Foxp3$^+$ and CD4$^+$Rorγt$^+$ T cells along with average percentage of CD4$^+$Foxp3$^+$ and CD4$^+$Rorγt$^+$ T cells at 30 days **C).** Representative images of percentages of CD4$^+$Foxp3$^+$ and CD4$^+$Rorγt$^+$ T cells along with average percentage of CD4$^+$Foxp3$^+$ and CD4$^+$Rorγt$^+$ T cells at 45 days. The above images are representative of one experiment and similar results were obtained in three different experiments with n=6 mice/group/experiment.

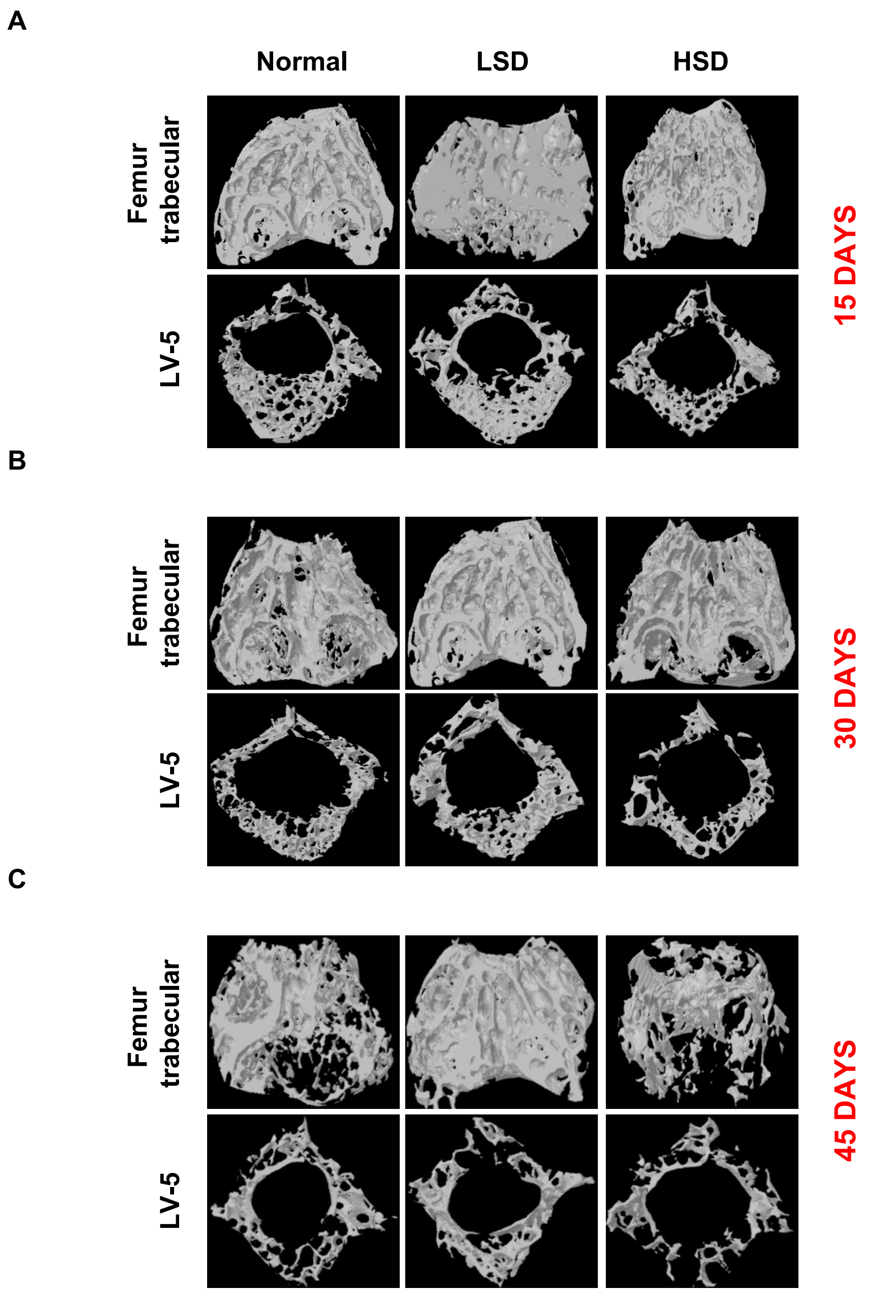

Supplementary Figure S2. High dietary salt significantly enhances bone loss at 45 days. Representative MicroCT images of normal, low salt diet (LSD) and high salt diet (HSD) mice lumbar vertebrae-5 (LV5) and femur trabecular at 15, 30 and 45 days of high dietary salt intake. **A).** MicroCT images at 15 days intake of high dietary salt. **B).** MicroCT images at 30 days intake of high dietary salt. **C).** MicroCT images at 45 days intake of high dietary salt. The above images are representative of one experiment and similar results were obtained in three different experiments with n=10 mice/group/experiment.

A

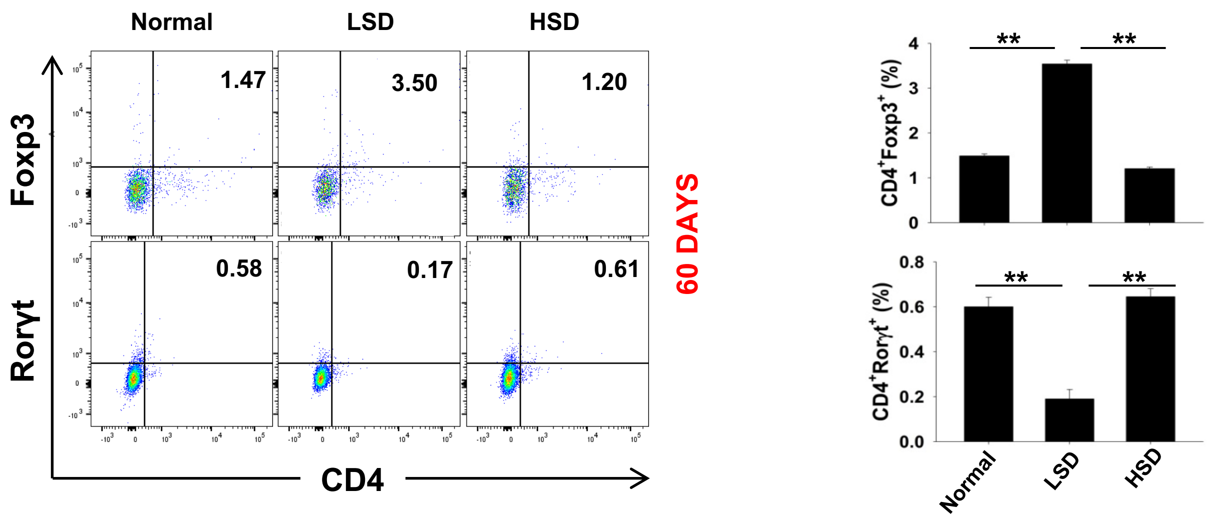

Supplementary Figure S3. Flow cytometry analysis of bone marrow cells at day 15 post removal of high/low salt diet and put back on normal salt diet. Mice were put back on a normal salt diet for 15 days after the end of 45 days high salt diet period. Cells from bone marrow (BM) of normal, low salt diet (LSD) and high salt diet (HSD) mice groups were isolated at the end of experiment, labelled and analysed by flow cytometry for percentage of $CD4^{+}Foxp3^{+}$ and $CD4^{+}Ror\gamma t^{+}$ T cells. Gating was first performed for $CD4^{+}$ and later analysed for the expression of $CD4^{+}Foxp3^{+}$ (Tregs) and $CD4^{+}Ror\gamma t^{+}$ (Th17) at 15 days post change of diet. **A).** Representative images of percentages of $CD4^{+}Foxp3^{+}$ and $CD4^{+}Ror\gamma t^{+}$ T cells along with average percentage of $CD4^{+}Foxp3^{+}$ and $CD4^{+}Ror\gamma t^{+}$ T cells at day 60 (45 days HSD/LSD + 15 days normal salt diet).

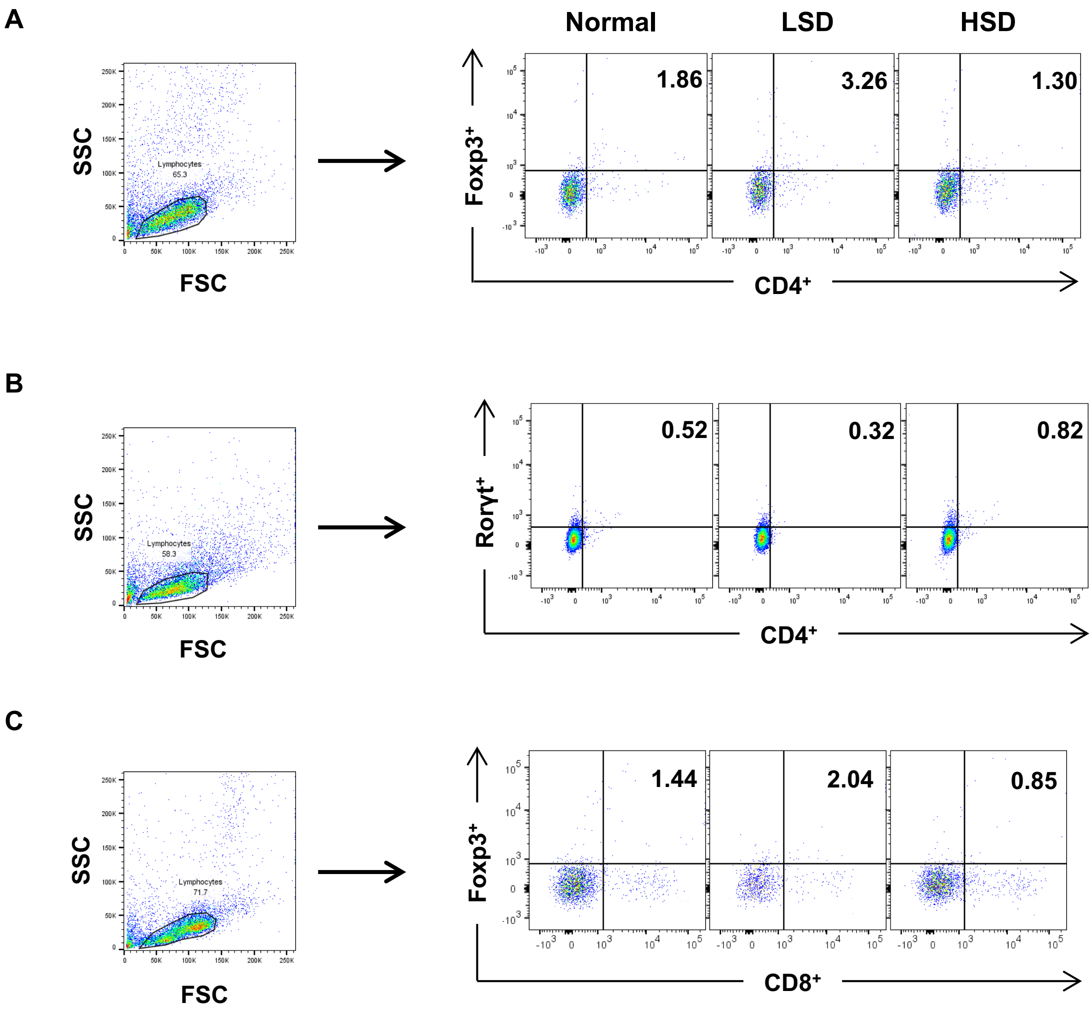

Supplementary Figure S4. Gating strategy for analyzing T cell population. Cells from bone marrow (BM) of normal, low salt diet (LSD) and high salt diet (HSD) mice groups were isolated at the end of experiment (day 45), labelled and analysed by flow cytometry for percentage of CD4$^+$Foxp3$^+$ and CD4$^+$Rorγt$^+$ T cells. Gating was first performed for CD4$^+$ and CD8$^+$ T cells and later analysed for the expression of CD4$^+$Foxp3$^+$ (Tregs), CD4$^+$Rorγt$^+$ (Th17), CD8$^+$Foxp3$^+$ (Tregs) cells. **A).** Percentage of CD4$^+$Foxp3$^+$ (CD4 Treg cells). **B).** Percentage of CD4$^+$Rorγt$^+$ (Th17 cells). **C).** Percentage of CD8$^+$Foxp3$^+$ (CD8 Treg cells). The above images are representative of one experiment and similar results were obtained in three different experiments with n=10 mice/group/experiment.

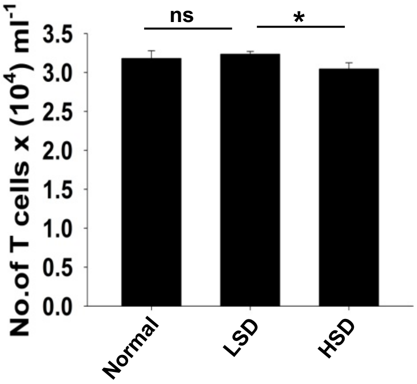

Supplementary Figure S5. T cell number count in bone marrow (BM) of normal, low salt diet (LSD) and high salt diet (HSD) at the end of 45 days. At the end of day 45 BM cells were flushed and T cells were isolated using already established protocol as discussed in material methods. The cells were counted using hemocytometer. Briefly 90ul of 0.4% trypan blue was mixed with 10ul of cell suspension and then 10ul of the resulting mixture is pipetted into a counting chamber. Cells were counted and multiplied with the percentage of T cells (obtained from flow cytometry) for total number of T cells (in 10^4 cells/ml) in different diet groups. Results are representatives of total number of T cells in 10^4 cells/ml. The above images are representative of one experiment and similar results were obtained in three different experiments with n=6 mice/group/experiment.
